# Clinical Characteristics of Patients with Gait Instability after MR-Guided Focused Ultrasound Thalamotomy

**DOI:** 10.5334/tohm.643

**Published:** 2021-10-21

**Authors:** Lauren M. Jackson, Timothy J. Kaufmann, Vance T. Lehman, Kendall H. Lee, Kai J. Miller, Anhar Hassan, Bryan T. Klassen

**Affiliations:** 1 Department of Neurology, Mayo Clinic, Rochester, MN, US; 2 Department of Radiology, Mayo Clinic, Rochester, MN, US; 3 Department of Neurosurgery, Mayo Clinic, Rochester, MN, US

**Keywords:** MR guided focused ultrasound thalamotomy, ventral intermediate nucleus, essential tremor, gait instability, neuropathy

## Abstract

**Background:**

MRgFUS thalamotomy is an incisionless procedure which effectively treats patients with tremor, although the procedure can result in adverse side effects including gait instability. By determining whether certain pre-existing conditions predispose patients to developing gait instability, we will be able to better counsel patients regarding risk of MRgFUS thalamotomy.

**Methods:**

All patients diagnosed with essential tremor, mixed tremor syndrome, or tremor predominant Parkinson disease who underwent MRgFUS thalamotomy at Mayo Clinic, Rochester between 2017 and 2020 were retrospectively reviewed. Baseline demographic and clinical data was extracted, and gait symptoms were compared pre- versus post-operatively.

**Results:**

Of 45 patients who underwent MRgFUS thalamotomy, 42 had at least one follow-up visit within twelve months and were included in the study. 39 patients had essential tremor, 1 had tremor predominant Parkinson disease, and 2 had mixed tremor syndrome. 19 out of 42 patients (45%) had gait decline. There were 10 (24%) females, and median age was 77.6 years (IQR 71.5–83.2). Older age was not correlated with gait decline (p = 0.82). Patients with a history of neuropathy and joint replacements were more likely to have gait decline after MRgFUS thalamotomy (p = 0.0099 and p = 0.0376). Patients with pre-existing gait aids were not more likely to have gait instability (p = 0.20).

**Conclusion:**

Patients who undergo MRgFUS thalamotomy for each of the tremor conditions, have an increased risk of experiencing gait decline, when there is a pre-procedure history of peripheral neuropathy, or joint replacement surgery. Older age or pre-existing gait aid use is not associated with worsened gait outcomes.

**Highlights:**

- Patients who undergo MRgFUS thalamotomy for tremor syndromes have a significantly increased risk of experiencing gait decline when there is comorbid peripheral neuropathy or joint replacement
- Older age or pre-existing gait aid use is not associated with worsened gait outcomes

## Introduction

MRgFUS thalamotomy is an incisionless and effective procedure for treatment of patients with essential tremor or tremor associated with Parkinson disease [1,2,3]. By using transducers which direct ultrasound energy to the ventro-intermedius nucleus (VIM) of the thalamus, an ablative lesion is created which results in improvement in contralateral tremor [4]. Although lesion location, size, and temperature can be monitored and adjusted based on clinical response during treatments, there may be persistent adverse events including paresthesia, dysarthria, imbalance, and hemiparesis [2,3,4,5]. Imbalance is one of the most common adverse side effects, occurring in 15–36% of reported patients with essential tremor (ET), and can be severe enough to require physical therapy or an additional gait aid in 11% of patients [2,3,6,7]. Conversely, conventional stereotactic radiofrequency thalamotomy is a lesion based therapy which is also associated with similar complications, including mild gait dysfunction (35%), although the inherent differences in ablative techniques limit the comparison [8]. Unfortunately the assessment of gait and balance intra-procedurally is not possible given the immobility of the patient within the head frame, and thus sonication parameters cannot be adjusted as they can for other side effects.

Prior studies have shown that the region of optimal therapeutic benefit for MRgFUS thalamotomy is localized to the border between VIM nucleus and ventral caudal nucleus of the thalamus [9]. More recently, tractography based imaging has been utilized for direct visualization of the dentatorubrothalamic tract (DRT) as an alternative target, as it is assumed that the tract coursing through the ventral thalamus corresponds to the VIM, and some studies have shown that targeting these tracts is as clinically beneficial as targeting the nuclei [9,10,11,12,13,14,15,16]. Although lesions inferolateral to the thalamus and within the dentatorubrothalamic tract have been associated with acute adverse gait effects, there is overlap noted in the areas associated with gait side effect and optimal therapeutic effect [9,17].

Although studies have shown that lesion size, maximum sonication temperature, and lesion location can affect the risk of gait disturbance, clinical features that influence the development of side effect after treatment are not well characterized [2,4,6,7,9]. In this study, we aim to identify clinical risk factors associated with the development of worsened gait stability after MRgFUS thalamotomy in patients with essential tremor, tremor predominant Parkinson disease, and mixed tremor syndrome.

## Methods

### Study Design

Upon institutional research ethics board approval, we retrospectively reviewed consecutive patients who underwent MRgFUS thalamotomy at Mayo Clinic, Rochester, MN between September 2017 and November 2020. Exclusion criteria included those who did not have available clinical follow-up or had a prematurely aborted procedure.

Patients were diagnosed with either essential tremor, tremor predominant Parkinson disease, or mixed tremor syndrome (combination of prominent resting and postural/kinetic tremor) after evaluation in DBS/neurostimulation clinic by a Movement Disorder specialist at Mayo Clinic, underwent comprehensive baseline multidisciplinary assessment, underwent electrophysiology testing to confirm organic tremor, and approval by DBS committee. All patients underwent at least one in person or virtual follow-up within 12 months after the procedure. Gait instability was defined as any subjective increase in imbalance or unsteadiness, an increase in number of falls, more consistent or upgraded use of gait aids, or if objective gait abnormalities were noted on post-procedural physical examination at follow-up. Transient gait instability was defined as symptoms persisting for less than 6 weeks. Other variables such as disease duration, past medical history or examination findings of neuropathy (such as stocking glove sensory deficit and lower extremity hyporeflexia), arthritis, joint replacements, and use of a gait aid prior to the procedure were collected.

### MR guided Focused Ultrasound Thalamotomy

Target selection was determined by first calculating the coordinate-based target (approximately 14 mm distance lateral to the AC-PC line (or 9.5–11 mm from the wall of the third ventricle) at a point 25% of the distance of the AC-PC line from the posterior commissure) and informed in most patients (n = 43) with DTI tractography of the dentatorubrothalamic tract (DRT), pyramidal tract, and somatosensory tract including the medial lemniscus. The lesion target was classified as more superior if it was centered 2 mm or more superior to the AC-PC line, and the target was classified as more lateral if it was more than 14 mm lateral to the midline. Our practice has targeted at the level of the AC-PC a slight amount (e.g. 2 mm) superior to it. Tractography was performed from the patient’s preoperative planning MRI and registered with preoperative T2-weighted imaging and the patient’s preprocedural CT. The final sonication target was chosen through coordinate-based targeting with input from DTI tractography, in order to ablate within the DRT (in V_im_, the ventral intermediate thalamic nucleus) while sufficiently avoiding sensory tracts (in V_c_, the ventral caudal thalamic nucleus) and the pyramidal tract.

The maximum sonication temperature was recorded. Post-operative imaging was reviewed, and lesion volume was calculated based on the immediate post-operative T2 weighted sequences (typically before there might be substantial surrounding vasogenic edema) by measuring the radius of one 2D slice of the lesion at the maximal diameter, and using a geometric ellipsoid formula to estimate volume (4/3 *π* (a/2 × b/2 × c/2)).

### Clinical assessment

All patients underwent clinical follow-up with a Movement Disorders subspecialist or Functional Neurosurgeon between six weeks to one year after MRgFUS thalamotomy. In those patients who were unable to travel in person for follow-up due to distance or pandemic related travel restrictions, a virtual or phone visit was completed instead. Symptom improvement, and side effects were elicited via history, and patients who were seen underwent a focused neurologic examination.

### Statistical analysis

JMP statistical software was used to perform statistical analyses, with statistical significance set at *p* < 0.05. Categorical data was analyzed using χ^2^ testing; continuous variables analyzed using the Wilcoxon signed rank test.

## Results

### Demographics

Between September 2017 and November 2020, 45 patients seen at Neurostimulation Clinic underwent MRgFUS thalamotomy for treatment of refractory essential tremor, mixed tremor syndrome, or tremor predominant Parkinson disease. Of the 45 patients reviewed, 42 had adequate follow-up and were included. Of the three not included, one was hospitalized for pneumonia and died several months after the procedure, which was thought to be unrelated, and two were not able to be contacted for follow-up. Of the 42 included, 10 (24%) were female and the median age was 77.6 years (interquartile range 71.5–83.2). 34 patients (81%) had the left VIM treated. 39 patients (93%) were diagnosed with essential tremor, two (5%) with mixed tremor syndrome, and one (2%) with tremor-predominant Parkinson disease (see ***Table 1*** for further details).

**Table 1:** Summary of tremor patients’ demographics and clinical data.


	ESSENTIAL TREMOR (39)	MIXED TREMOR SYNDROME (2)	TREMOR PREDOMINANT PARKINSON DISEASE (1)

**Age of patient, years**	78 (72–83)	76 (70.8–82)	68

**Female sex (%)**	10 (26)	0 (0)	0 (0)

**Left thalamotomies (%)**	32 (82)	1 (50)	1 (100)

**Neuropathy**	9 (23)	1 (50)	0 (0)

**Arthritis**	9 (23)	2 (100)	0 (0)

**Joint replacement**	5 (13)	1 (50)	0 (0)

**Maximal temperature achieved during MRgFUS °C**	59 (57–61)	60	57

**Lesion volume, mm^3^**	140 (112–204)	154 (73–235)	224

**Gait dysfunction after MRgFUS (%)**	17 (44)	1 (50)	1 (100)

**Persistent gait dysfunction after MRgFUS (%)**	12 (31)	1 (50)	1 (100)

**Severe gait dysfunction after MRgFUS (%)**	5 (13)	0 (0)	1 (100)


Values are median (interquartile range). Number of patients included: screened = 45; included = 42.

### Clinical Characteristics

Of the patients who underwent MRgFUS thalamotomy, 33 patients had one follow-up visit, and 9 patients had two follow-up visits after the procedure. The median follow-up time was 6 months (IQR 3–8). 20 patients had an in-person follow-up visit, and 22 patients had a virtual follow-up either via video or phone call. Four patients (10%) had pre-procedural gait dysfunction and required use of a gait aid prior to the procedure. One patient had a history of femoral neuropathy which necessitated occasional cane use, one had a history of macular degeneration for which he used a cane, one had a history of sensory axonal polyneuropathy for which he had a cane and walker for longer distances, and one had lower limb tremor for which she used a cane occasionally.

Of the patients who underwent treatment, 19 (45%) had worsened gait stability by either history or exam after the procedure. Five (12%) of patients had transient worsening of gait which resolved within six weeks after the procedure; and 14 (33%) had persistent gait decline at follow-up between six weeks to one year after the procedure. Of patients who had persistent gait decline at follow-up, six (15%) required a higher level of gait assistance (walker or cane) than their pre-procedural baseline, suggestive of severe gait dysfunction compared to their baseline. Ten patients had pre-existing neuropathy, and eight (80%) had worsened gait instability after MRgFUS thalamotomy (p = 0.0099), which was persistent in seven of the 10 patients (p = 0.0057), and severe enough to require an increased level of gait assistance in four patients (p = 0.0156). A history of joint replacement was associated with worsened gait instability after MRgFUS (p = 0.0376). A history of requiring a gait aid prior to the procedure was not associated with a worsening of gait post-procedure (p = 0.20).

Sex, diagnosis of tremor syndrome, and history of arthritis were not associated with gait decline by either history or exam after MRgFUS thalamotomy (p = 0.66, p = 0.44, p = 0.15 respectively). The median disease duration was 25 years (12.5–41) and was not associated with an increased risk of developing gait dysfunction (*p* = 0.5158). The single patient with tremor-predominant Parkinson disease, and one of the two mixed tremor syndrome patients had gait decline. 14 (36%) patients reported complete resolution in tremor at follow-up; this was not associated with gait decline compared to those with residual tremor at follow-up (p = 0.30).

### Imaging Characteristics

**Figure 1:**
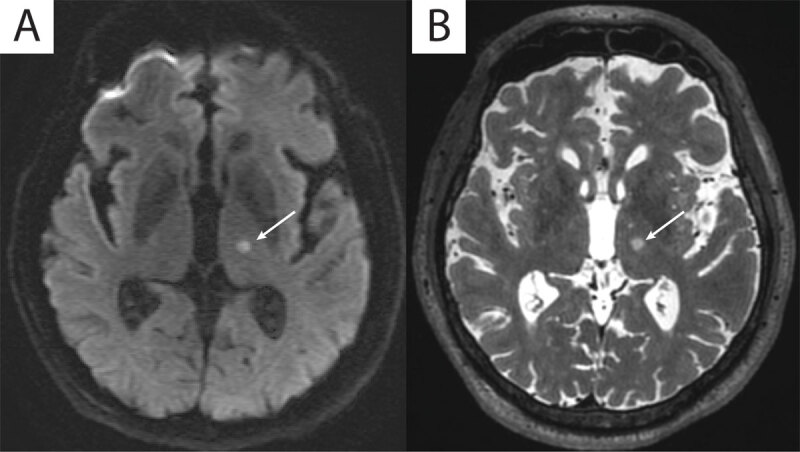
An 80 year-old patient with a typical immediate post-procedural imaging result depicting a spheroid-shaped lesion in the left ventral thalamus with restricted water diffusion. **A.** diffusion weighted imaging and **B.** hyperintense signal on axial T2-weighted imaging.

The median lesion volume (mm^3^) was 140 mm^3^ (IQR 112–211) and median maximum sonication temperature was 59 degrees Celsius (IQR 57–60) were not significantly associated with worsened gait stability after MRgFUS thalamotomy (p = 0.25 and p = 0.88 respectively). ***Figure 1*** depicts a typical imaging result. Initial planned targets which were more inferior (less than 2 mm superior to the AC-PC line) and more lateral (more than 14 mm lateral to the midline) were not associated with increased risk of gait decline after MRgFUS thalamotomy (p = 0.54, p = 0.30).

## Discussion

Our findings confirmed prior studies showing that patients who undergo unilateral MRgFUS thalamotomy for treatment of tremor syndromes have a significant risk of gait decline (45%) which remained persistent in 33% of our patients [1,2,3,4,6,7,9]. Prior studies have outlined that the risk of severe gait disturbance is relatively low (1%), defined as being unable to perform activities of daily living; in our cohort, we defined severe gait dysfunction as enough to require an increased level of gait aid, which occurred in 14% of patients at the first follow-up visit [7]. This further confirms the need to counsel patients regarding risk of significant gait decline versus benefit of proceeding with MRgFUS thalamotomy for treatment of tremor. In addition, when consideration is made for bilateral MRgFUS thalamotomy if this becomes available in the future, the safety profile should be cautiously considered in patients with increased risk of gait decline.

Although well-described in essential tremor, gait dysfunction has not been well-described in other tremor syndromes treated with MRgFUS thalamotomy. Gait dysfunction was observed with mixed tremor syndromes and tremor predominant Parkinson disease as well, although comprised very few cases in our total cohort. This finding suggests that an increased risk of postural instability present in patients with Parkinson disease may be influencing the development of gait dysfunction, however there were not enough subjects in our study to definitely make this conclusion and needs to be verified in larger studies of MRgFUS for these disorders [18].

**Figure 2:**
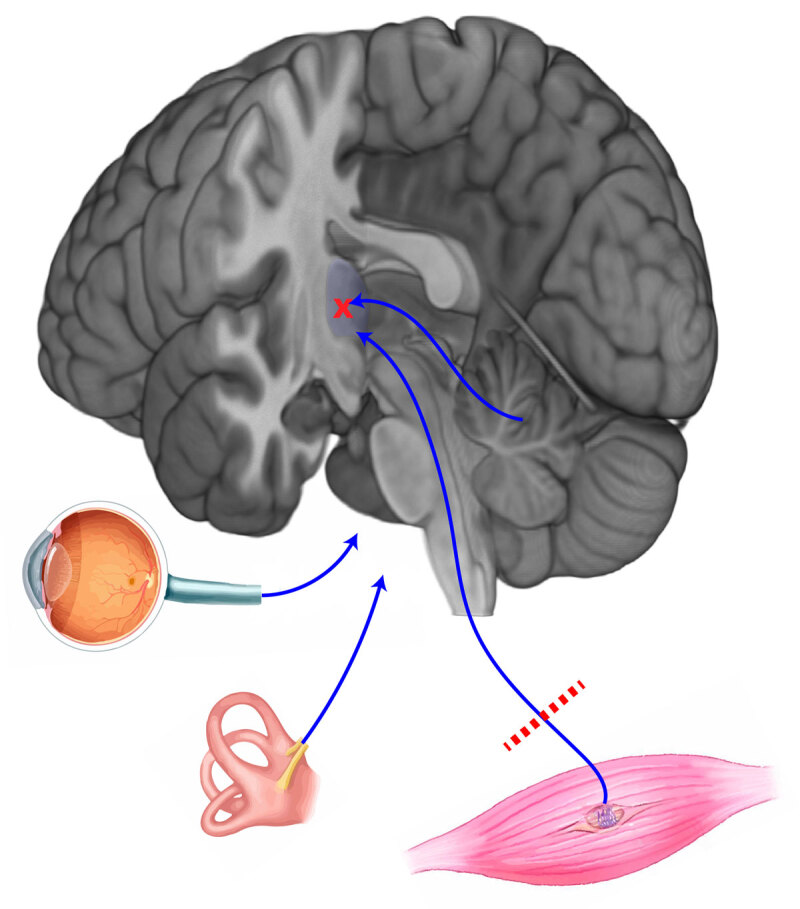
Dysfunction of visual, proprioceptive, vestibular, or cerebellar input can affect balance; in patients with multiple systems disrupted (e.g., proprioceptive and cerebellar) balance is more affected.

In our study, we found that patients with a history of neuropathy are more likely to have gait decline after MRgFUS thalamotomy than those without neuropathy. This suggests that impairment along the proprioceptive pathway combined with cerebellar dysfunction due to a lesion within the dentatorubrothalamic tract leads to increased susceptibility to developing gait dysfunction (see ***Figure 2***). Hence, all patients who are being considered for MRgFUS thalamotomy should be screened for neuropathy at the pre-procedure clinic visit and need to be thoroughly counselled with regards to the significant risk of gait decline. We also found that joint replacement was associated with gait decline which could be associated with the loss of proprioceptive feedback from the artificial joint, as the patients with arthritis did not have a risk of gait decline and still retain the natural joint.

Gait dysfunction is known to increase with age; however, in our cohort we did not find that age was significantly associated with risk of gait decline after the procedure [19]. Given that the tremor syndromes being treated with MRgFUS thalamotomy instead of deep brain stimulation are most prevalent among the elderly, it is possible that the relatively narrow age range seen in our cohort masked the effect that age can have on gait dysfunction. We also found that patients with pre-existing gait aids were not more likely to develop worsening gait instability after the procedure. In these patients, the consistent use of the gait aid may be masking subtle differences which prevent any difference from being noted by the patients or their providers at follow-up.

Although larger lesions and higher sonication temperatures have been shown to be associated with increased risk of adverse effects, in our study we did not find that lesion volume or maximum sonication temperature was significantly correlated with worsened gait stability after MRgFUS thalamotomy. This may be secondary to a narrow median lesion volume and maximum sonication temperature range which did not vary significantly between patients. Prior studies have shown that lesion location in the inferolateral thalamus is more likely associated with acute gait dysfunction, but in our population, lesions that were considered more lateral and inferior were not associated with increased risk of gait dysfunction [9].

Strengths of this study include the large MRgFUS thalamotomy patient cohort. Previous studies have described the consequence of gait instability after focused ultrasound; however to our knowledge no studies have yet demonstrated the correlation of clinical characteristics such as neuropathy, mechanical joint disease, as well as how age and prior need for gait aids does not lead to heightened risk of worsened gait stability after the procedure.

Limitations include the retrospective nature of the study. Follow-up time was variable, in some cases being short-term. The degree of proprioceptive loss was unable to be assessed given the retrospective nature of the study and variability of clinical documentation with regards to the neurologic exam. Due to the nature of virtual follow-up secondary to the COVID-19 pandemic, gait dysfunction was assessed on a clinical basis and a formal gait dysfunction rating scale was not utilized thus limiting the ability to quantify degree of gait disability. Lesion location was estimated based on peri-procedure tractography in relation to the atlas-based targeting and did not include peri-procedural adjustments based on lack of efficacy or side effect.

Future directions should include a larger prospective longitudinal cohort to assess the impact of risk factors such as age and other clinical characteristics on the development and progression of adverse effects with long term follow-up after the procedure. A larger double blind controlled study with blinded evaluations from neurologists not aware of which patients received focused ultrasound versus sham procedure examining both essential and non-essential tremor patients are also needed to understand complications of the procedure and determine risk factors associated with adverse effects in this patient population. Further study of specific technical factors which may contribute to gait dysfunction, such as precise ultrasound-induced lesion location in or relative to V_im_, is also warranted. It is not certain if spatial adjustment of targeting within the ventral thalamus can continue to yield good tremor control while avoiding significant risk of gait dysfunction.
